# New Appliances and Things Medical

**Published:** 1898-01-15

**Authors:** 


### NEW APPLIANCES AND THINGS MEDICAL.

[We shall be glad to receive, at our Office, 28 & 29, Southampton Street, Strand, London, W.C., from the manufacturers, specimens of all new preparations and appliances which may be brought out from time to time.]

### VOLORA TEA. W. K. BRAND.

(United Kingdom Tea Company, 21, Mincing Lane, E.C.)

We have examined a sample of Volora tea, and find it soft and agreeable to the palate. Its comparative freedom from astringent action on the mucous membrane of the mouth, even when strongly infused, is a safe indication that it can have no deleterious tanning action on the coats of the stomach when in ordinary strength. Our practical experience of this tea fully coincides with the results obtained by well-known chemists from analytical examination, and we unhesitatingly recommend it to the notice of those who cater for the sick.

